# Genome-wide association scan and phased haplotype construction for quantitative trait loci affecting boar taint in three pig breeds

**DOI:** 10.1186/1471-2164-13-22

**Published:** 2012-01-13

**Authors:** Vivi R Gregersen, Lene N Conley, Kirsten K Sørensen, Bernt Guldbrandtsen, Ingela H Velander, Christian Bendixen

**Affiliations:** 1 Department of Molecular Biology and Genetics, Faculty of Science and Technology, Aarhus University, P.O. Box 50, DK-8830 Tjele, Denmark; 2 Danish Agriculture & Food Council, Pig Research Centre, Axeltorv 3, 1609 Copenhagen V, Denmark

## Abstract

**Background:**

Boar taint is the undesirable smell and taste of pork meat derived from some entire male pigs. The main causes of boar taint are the two compounds androstenone and skatole (3-methyl-indole). The steroid androstenone is a sex pheromone produced in the testis of the boars. Skatole is produced from tryptophan by bacteria in the intestine of the pigs. In many countries pigs are castrated as piglets to avoid boar taint, however, this is undesirable for animal welfare reasons. Genetic variations affecting the level of boar taint have previously been demonstrated in many breeds. In the study presented in this paper, markers and haplotypes, which can be applied to DNA-based selection schemes in order to reduce or eliminate the boar taint problem, are identified.

**Results:**

Approximately 30,000 SNPs segregating in 923 boars from three Danish breeds; Duroc, Landrace, and Yorkshire, were used to conduct genome wide association studies of boar taint compounds. At 46 suggestive quantitative trait loci (QTL), 25 haplotypes and three single markers with effects were identified. Furthermore, 40% of the haplotypes mapped to previously identified regions. Haplotypes were also analysed for effects of slaughter weight and meat content. The most promising haplotype was identified on *Sus scrofa *chromosome 1. The gain in fixed effect of having this haplotype on level of androstenone in Landrace was identified to be high (1.279 μg/g). In addition, this haplotype explained 16.8% of the phenotypic variation within the trait. The haplotype was identified around the gene *CYB5A *which is known to have an indirect impact on the amount of androstenone. In addition to *CYB5A*, the genes *SRD5A2*, *LOC100518755*, and *CYP21A2 *are candidate genes for other haplotypes affecting androstenone, whereas, candidate genes for the indolic compounds were identified to be *SULT1A1 *and *CYP2E1*.

**Conclusions:**

Despite the small sample size, a total of 25 haplotypes and three single markers were identified including genomic regions not previously reported. The haplotypes that were analysed showed large effects on trait level. However, little overlap of QTL between breeds was observed.

## Background

Boar taint is defined as an unpleasant flavour and odour of porcine meat. It is mainly two compounds that contribute to boar taint. 3-methylindole, also called skatole, is a metabolite of tryptophan metabolism and is produced by intestinal bacteria in the hindgut of the pigs [1]. When cooking, skatole released from fat, gives the meat a faecal-like smell. Androstenone, a more urine smelling compound is also accumulated in adipose tissue. It is biosynthesised from cholesterol in boar testes (reviewed in [2]). It was previously found that levels of both compounds are linked to sexual maturity of male pigs [3] and that there is a relationship between their metabolisms [4]. It has been shown that androstenone, at least *in vitro*, can inhibit metabolism of skatole by *CYP2E1 *[5,6]. The heritability of levels of both compounds is substantially ranging from 0.23 to 0.55 for skatole and from 0.49 to 0.67 for androstenone in the Landrace and Duroc breeds [3,7] but the level of accumulated compound varies between breeds [8].

In most pig-producing countries castration is used to avoid boar taint. However, castration of male pigs also results in reduced feed conversion efficiency as well as in a reduction in carcass trait values (reviewed in [9]). Ongoing research in the area of dietary composition in relation to biosynthesis of skatole shows that certain carbohydrates influences the micro-flora and hence skatole production. An increase in faecal wet and dry weight is observed when the diet comprises unsaturated carbohydrates [10] and the consequent decrease in the transit time in the intestine has an influence on the level of absorbed skatole [11]. However, dietary composition does not affect the level of androstenone. Immunocastration by Improvac™ in relation to levels of both androstenone and indolic compounds has shown promising results [12]. The sexual development is inhibited by disrupting the hypothalamic-pituitarygonadal axis. As a result, the development of testes and hormone synthesis is hindered thereby suppressing androstenone synthesis. Furthermore, lower levels of testicular steroids in immunocastrates accelerate the metabolic clearance of indolic compounds [13]. However, as the second vaccination has to be applied to rather large animals a need for a more practical procedure is recommended [14]. The cost of vaccinating the boars and the loss of their preferred carcass traits and feed consumption is still an issue.

Enzymes and regulatory proteins relating to both androstenone and skatole concentrations have been reviewed in several papers [2] and [15]. Androstenone along with other steroid hormones are synthesized from pregnenolone or progesterone through the formation of 5,16-androstadien-3β-ol or 4,16-androstadien-3-one, respectively. In the first process two genes, *CYB17A1 *and *CYB5*, are involved in this synthesis [16]. It is controlled by the neuroendocrine system which also regulates other testicular steroids. In the second process the gene *CYP21 *is involved [2]. Both skatole and androstenone accumulates in fat and the degradation takes place in the liver through both phase I and phase II metabolism before excretion. The hepatic metabolism in phase I degradation of androstenone is mediated by 3-hydroxysteroid dehydrogenase enzymes [17] and influenced by the presence of *NADH *and *NADPH *as cofactors [18] whereas skatole is controlled by *CYP2E1 *[5,19-21], *CYP2A *[21] and aldehyde oxidase [22]. Phase II of the hepatic metabolism involves sulfotransferases [23] and uridine diphosphate-glucuronosyltransferase enzymes [20,24-27]. In addition, nuclear receptors are believed to play an important role in regulating the expression of genes involved in androstenone and skatole metabolism [28].

Different approaches have been made to link candidate genes to boar taint. One approach is to locate a genomic region associated with the trait and hence use the candidate gene approach to identify the gene or genes in question. A number of quantitative trait loci (QTL) and association studies involving both purebred and crossbred pedigrees have been conducted and genes have been located within some of these QTL regions [7,29-33]. Another method is to use gene expression analysis where differentially expressed genes in different tissues have revealed new candidate genes [34-36]. Some of the candidate genes that are likely to have an impact on the level of boar taint have been summarised by Moe *et al*. (2009) [37] who performed an association study of single nucleotide polymorphisms (SNPs) located in the candidate genes. Recently, Grindflek *et al*. (2010) [38] analysed differential expression of 15 selected candidate genes found in testes of animals from the Norwegian Duroc and Landrace with high and low levels of androstenone. Their investigation revealed that 12 of the genes were differentially expressed. Among these only *CYB5A *was cis-regulated.

Using SNPs genotyped in three different Danish pig breeds using the porcineSNP60 BeadChip we aim to identify genomic regions associated with skatole, androstenone, indole as well as the S/I index, which is a skatole equivalent measured by the slaughterhouse containing both skatole and indole. A comparison of the QTL identified within and between breeds in relation to the different traits will be conducted. By implementing a new definition of QTL haplotypes, where SNPs are included if they are highly associated, we aim to increase the probability of discovering markers linked to QTL. The ultimate objective is to identify haplotypes that can be used directly in a marker assisted selection (MAS) strategy to breed for preferred levels of the boar taint traits. Finally, candidate genes associated to the QTL regions will be discussed.

## Results

### Genotyping and data validation

A total of 923 animals were genotyped on the Illumina porcineSNP60 BeadChip resulting in 27,451, 30,396 and 30,497 segregating SNPs in Duroc (D), Danish Landrace (L) and Yorkshire (Y), respectively. All samples had a call rate above 0.95 and all SNPs selected for further analysis had a call frequency above 0.96. All SNPs were validated with regard to Hardy-Weinberg proportions (HWP) and minor allele frequency (MAF). A total of 2,528 SNPs in D, 1,733 SNPs in L and 120 SNPs in Y were discarded due to low HWP p-values (p < 0.001). In addition, a total of 18,449, 15,303 and 15,399 SNPs in D, L and Y, respectively, were removed due to low MAF. The number of tag SNPs [39] for each of the three breeds were 11,494, 13,469 and 13,121 for D, L and Y, respectively. The genome-wide Bonferroni significance levels (p = 0.05) for the three breeds were found to be p = 4.2 × 10^-6^, p = 3.5 × 10^-6 ^and p = 3.7 × 10^-6^, respectively. Chromosome-wide Bonferroni significance levels (p = 0.05) were calculated from the amount of tag SNPs representing the different chromosomes. The levels varied between p = 8 × 10^-5 ^and p = 3 × 10^-5 ^depending on the number of segregating markers in the breed and the size of the chromosome. As expected from previous findings the traits showed great variation between the breeds (Table 1). The phenotypic traits were adjusted using log base 10 to obtain a Gaussian distribution. Compared to previous studies [8], the skatole level within the L population is significantly higher in the present study.

**Table 1:** Statistical properties of the boar taint traits in the three breeds*.

Trait (μg/g)	N	Mean	SD	Min	Max
Duroc					
Skatole/Indole	240	0.0829	0.139	0.014	1.83
Skatole	179	0.0637	0.099	0	0.745
Androstenone	180	3.5394	2.511	0.07	19.83
Indole	179	0.0533	0.0374	0.012	0.213
Danish Landrace					
Skatole/Indole	331	0.1956	0.1826	0	1.92
Skatole	258	0.2537	0.2029	0	2.551
Androstenone	259	1.318	1.1143	0.16	8.83
Indole	258	0.0492	0.0596	0.008	0.688
Yorkshire					
Skatole/Indole	217	0.0678	0.0449	0	0.43
Skatole	132	0.0446	0.0486	0	0.317
Androstenone	132	0.952	0.9138	0.13	6.9
Indole	132	0.027	0.0183	0.007	0.16

*Number of boars (N), Mean trait value and standard deviation (SD), minimum (Min) and maximum (Max) value of the trait

### Quantitative trait loci (QTL) analysis

The porcine genome was scanned by a simple approach to identify regions for further analysis. By using a simplified analysis it is expected to detect a number of spurious QTL in addition to the true QTL. The QTL identified by this scan should be seen as suggestive candidate regions. A total of 46 suggestive QTL were identified within the three porcine breeds. In total, 17 showed associations to androstenone, 12 to skatole, eight to the combined trait of both skatole and indole (S/I index) and nine to indole. Initially, four QTL were identified to be significant at the 5% genome-wide Bonferroni level, and two at the 1% level. However, only 28 were confirmed by the haplotype and single marker analysis (Table 2).

**Table 2:** Chromosome wide significant boar taint QTLs confirmed by a haplotype analysis mapped to Sscrofa10.2 in different breeds.

		QTDT	QFAM		
				
SSC^#^	Trait	p-value	emperical p-value	Peak (Mb)	Interval (Mb)
Duroc					
3	log Skatole	2.0E-05'	8.0E-05	19.615	16.518-20.52
3	log Androstenone	8.0E-05	4.0E-05	120.04	113.99-122.62
5	log S/I-index	1.0E-05	2.0E-05	18.728	18.728-18.962
6	log Androstenone	1.0E-05	3.0E-05	75.721	75.721-76.450
10a	log Indole	4.0E-05	NS	7.961	7.793-18.265
10b	log Indole	2.0E-05	NS	62.985	52.066-63.550
12	log Androstenone	1.0E-05	2.0E-05	41.225	41.209-46.114
14	log Androstenone	6.0E-05	6.0E-05	19.421	19.421-21.826
Landrace					
1	log Androstenone	3.0E-05	NS	164.281	164.281-166.282
2a	log Androstenone	2.0E-05	6.0E-05	98.469	85.522-106.398
2b	log Androstenone	2.0E-05	4.0E-05	142.568	142.568-144.814
5	log S/I-index	6.0E-06	4.0E-05	87.29	62.442-88.563
6	log Indole	7.0E-06	3.0E-05	134.996	134.141-140.591
7	log Androstenone	1.0E-06*	1.0E-05	40.375	2.451-40.421
11	log Indole	4.0E-05	4.0E-05	87.549	single SNP
12	log Androstenone	5.0E-05	4.0E-05	45.898	single SNP
14	log S/I-index	3.0E-05	3.0E-05	152.887	149.608-153.745
14	log Indole	2.0E-06*	1.0E-05	151.637	150.832-153.786
15	log Indole	4.0E-05	4.0E-05	17.709	2.724-17.767
Yorkshire					
6a	log S/I-index	NS	1.0E-05	76.056	75.839-80.408
6b	log S/I-index	NS	2.0E-05	157.089	155.897-157.734
7	log S/I-index	3.0E-05'	1.0E-05	99.096	98.947-101.548
8	log S/I-index	NS	2.0E-05	38.293	35.328-40.051
9	log Skatole	7.0E-06	1.0E-05	30.292	15.055-30.539
11b	log Androstenone	8.0E-06	7.0E-05	75.129	78.672-80.006
12	log Androstenone	2.0E-05	7.0E-05	37.443	35.941-45.383
14	log Androstenone	5.0E-06	2.0E-05	138.164	single SNP
14	log Indole	6.0E-07**	1.0E-05	152.48	149.326-153.593

**,* = p < 0.01, p < 0.05 genome-wide significant, ^# ^= *Sus scrofa *chromosome

' = QTDT on none log transformed trait, NS = not significant at the 5% Bonferroni level

### Haplotype analysis and QTL effect

The phased haplotype analysis for each of the suggestive QTL was conducted on SNP-sets containing between four and 24 SNPs identified within a single breed (Table 3, Additional File 1). The haplotype analysis identified between two and 14 haplotypes with a frequency of more than 1.5% accounting for 58.7% to 99.4% of the data. The most frequent haplotype identified by the haplotype algorithm was analysed for an additive fixed effect by a mixed model taking into account the sire, dam, season, herd and pen random effects. In 24 out of 43 cases the most frequent haplotype had a significant fixed effect on the level of the trait. In one case the second most frequent haplotype was analysed as well. This was done as mean value represented by the second most frequent haplotype showed a larger deviation from the total mean than the most frequent. In addition, the number of animals carrying the two haplotypes was similar. The frequencies of the tested haplotypes were between 19.8% and 82.8%. In seven QTL the fixed effect of the haplotype had a negative value. This was true when the analysed haplotype would increase the boar taint level.

**Table 3:** Haplotype statistics and analysis of suggested QTL regions.

						Haplotype analysis	
						
SSC^#^	Trait	HF	SNP-set	Haplotypes^$^	Percent^$^	Significance	Fixed effect (μg/g)
Duroc							
3	log Skatole	68.9	10	5	93	0.05023	0.022
3	log Androstenone	33.7	12	10	97.4	0.03365 *	0.844
5	log S/I-index	81.6	5	2	99.4	0.00304 **	0.015
6	log Androstenone	56.1	4	4	99.4	2.106e^-4 ^***	0.775
10a	log Indole	55.4	10	13	78	6.92e^-4 ^***	0.012
10b	log Indole	34.1	15	12	77.2	0.0225 *	0.008 (0.003*)^a^
12	log Androstenone	51.5	9	9	93.4	1.027e^-4 ^***	1.292
14	log Androstenone	82.8	12	4	94.9	7.4e^-3 ^**	1.183
Landrace							
1	log Androstenone	19.8^S^	6	8	99.2	3.35e^-4 ^***	1.279
2a	log Androstenone	23.4	7	14	85.7	6.48e^-3 ^**	0.838
2b	log Androstenone	54.9	6	6	94.2	0.02133 *	1.172
5	log S/I-index	44.5	10	9	79.6	6.138e^-3 ^**	-0.031
6	log Indole	27.6	11	10	89.3	0.01616 *	-0.012
7	log Androstenone	38.6	15	13	58.7	8.094e^-3 ^**	1.240
14	log S/I-index	52.4	24	9	92.2	3.86e^-5 ^***	-0.038 (-0.011***)^a^
14	log Indole	30	24	7	93.3	8.815e^-5 ^***	-0.013 (-0.004***)^a^
15	log Indole	48.1	18	7	96	5.451e^-3 ^**	-0.056
Yorkshire							
6a	log S/I-index	73.9	15	3	95.8	4.706e^-5 ^***	0.015 (0.004***)^b^
6b	log S/I-index	30.6	10	7	94.4	0.01399 *	0.009
7	log S/I-index	44.8	16	5	97.2	3.538e^-3 ^**	0.013
8	log S/I-index	63	11	5	97.2	1.741e^-3 ^**	0.013 (0.003**)^a^
9	log Skatole	33.5	11	10	90.5	0.03686 *	-0.012
11b	log Androstenone	57.1	5	6	98.4	0.0125 *	0.838 (0.059**)^a^
12	log Androstenone	61	16	10	94.3	0.03257 *	0.827
14	log Indole	43.8	12	8	95.1	1.028e^-3 ^**	-0.007

***,**,* = p < 0.001, 0.01, 0.05, ^# ^= *Sus scrofa *chromosome

HF = Haplotype frequency of the most common haplotype,^S ^= The second most common haplotype

^$ ^= Amount of haplotypes with a frequency above 1.5%

^a^,^b ^= Test including slaughter weight, slaughter weight and meat content as covariate

In three cases the identified QTL was explained by a single SNP. In these cases the genotype was analysed by the same additive mixed model as haplotypes were. When analysing the effects of being a carrier of the most frequent allele within the SNPs, the SNP in L affecting indole on SSC 11 showed a fixed effect of 0.004 μg/g (p < 0.05), whereas, the fixed effect of the two SNPs affecting androstenone on SSC 12 in L and on SSC 14 in Y were identified to be 0.945 μg/g (p < 0.001) and 0.126 μg/g (p < 0.001), respectively.

### Body trait analysis

Since meat content and slaughter weight could potentially be affected by some of the genes that affect boar taint compounds it was decided not to include these two traits in the mixed model. Instead, we examined the derived haplotypes with respect to both meat content and slaughter weight. For six out of the 25 haplotypes, a significant effect between slaughter weights and the haplotype was also found. In one of these cases the meat content was also affected by the haplotype differentiation (Table 4). The six haplotypes affected by the slaughter weight were further analysed to see the effect of the weight on the trait. This was done by the mixed model including the slaughter weight and in one case also the meat content as covariates. As expected the fixed effect of the haplotype was reduced for all haplotypes investigated (Table 3). None of the single markers were affected by the body traits.

**Table 4:** Influence of body traits on haplotypes.

	Slaughter weight		Meat content	
	
SSC^#^	Significance	Fixed effect (kg)	Significance	Fixed effect (%)
Duroc				
10b	0.0270 *	1.199		
Landrace				
14	8.277e^-3 ^**	1.305		
14	8.908e^-3 ^**	1.427		
Yorkshire				
6a	6.929e^-3 ^**	1.339	0.0377 **	0.273
8	7.207e^-3 ^**	1.393		
11b	0.0469 *	1.162		

**,* = p < 0.01, p < 0.05, # = *Sus scrofa *chromosome

## Discussion

This GWAS identified 46 chromosomal regions affecting the four analysed boar taint traits. The highly associated SNPs around the QTL peak were used to identify haplotypes describing the genetic variation affecting the QTL. The SNP-sets were analysed by use of a probability based phasing algorithm implemented to infer haplotypes. Of the 46 suggestive QTL, 28 were confirmed using a haplotype analysis by contrasting the most abundant haplotype to the rest of the haplotypes with an additive effects model. The QTL and haplotype analysis was performed in the three Danish breeds Duroc, Landrace, and Yorkshire, separately. A total of 10 haplotypes were found to significantly affect androstenone, two to affect pure skatole, six to affect pure indole and seven to affect a skatole equivalent (S/I index) containing both skatole and indole. In addition, slaughter weight and meat content were analysed and sometimes found to be significantly affected by the QTL haplotype. By including slaughter weight and meat content as covariates in the mixed model the fixed effects for the haplotypes were adjusted. The sample size of each breed was limited (132 to 331). Therefore QTL discovered here should be validated using larger samples of the same populations. However, the number of half sib families analysed resembles the number found in a previous study [33]. Also as at most two offspring from each nucleus family were included, we are convinced that a large part of the genetic variation within each breed was represented in the data. In addition, by introducing haplotypes capturing the total genetic variance within the QTL region, and not only within haplotype blocks split by the four gamete rule as is a more conventional approach [40], it was possible to select and combine information from markers likely to be in strong linkage disequilibrium with whatever regulates the trait. These two things in combination might explain the relatively large effects found in this study.

### QTL analysis

Previous studies of QTL affecting androstenone in fat identified genomic regions on SSC 1, 2, 3, 4, 5, 6, 7, 9, 10, 11, 13, 14, 15 and 18 [7,29,32,33]. In the current study, a substantial number of genome regions affecting fat androstenone were identified in the three purebred populations analysed. The affected chromosomal regions that were confirmed by the haplotype analysis were located on the following chromosomes: 2, 3, 5, 6, 7, 11, 12 and 14.

Few studies have been conducted to identify genome regions affecting skatole in fat. However, the two most recent studies by Grindflek *et al*. (2011) [7,33] detected QTL regions affecting both skatole and indole on nine different chromosomes. In total, ten chromosomes have been identified to harbour QTL affecting skatole and the SSC locations are 1, 2, 3, 5, 6, 7, 10, 11, 13 and 14 [7,29,30,33]. In the current study, regions affecting skatole were identified on SSC 3 and 9. In relation to indole, the current study identified seven chromosomal regions to be affected. These were located on SSC 6, 10, 11, 14 and 15. In addition, segmental regions identified to be affected by the S/I index were found on SSC 5, 6, 7, 8 and 14.

### Analysis within and between breeds

The QTL affecting the indole and S/I index trait, identified on SSC 14 in L, was the only QTL found to overlap within a breed. As indole is part of the S/I index an overlap in QTL region between these two traits is not surprising. Overlaps were also observed between breeds, however, mostly within the same trait. The QTL on SSC 12, found to affect androstenone, was segregating in all breeds, but it was only explained by a single SNP in L. An overlap could also be observed between the QTL for androstenone in D and the QTL for the S/I-index in Y on SSC 6. In addition, the QTL on SSC 14 previously described in L also overlap a QTL within the same region in Y. The relatively small overlap in QTL could be expected based on the knowledge of the different distribution of the boar taint traits observed within the three breeds and in relation to possible fixed alleles.

### Haplotype analysis in relation to androstenone

A suggestive QTL reducing the level of androstenone was observed in D on SSC 3. It was confirmed by the haplotype analysis. The most frequent haplotype showed a fixed effect of 0.844 μg/g, which is relatively small compared to other effects identified in the present study. However, as the frequency of the haplotype is around 34%, it is possible to increase the population frequency by selection. One of the genes previously identified as being involved in androstenone biosynthesis was located within the QTL region, i.e., the gene *ST5AR2 *(steroid 5-alpha-reductase 2) also called *SRD5A2*. A number of steroids are catabolised by this enzyme along with *SRD5A1 *into their 5α-reduced metabolites [41]. In addition, SRD5A2 is also involved in androstenone formation by catabolising the final step from 4,16-androstadien-3-ene [2]. Variation of SNPs within the SRD5A2-3'UTR has previously been shown to be associated to plasma levels of androstenone in a Norwegian Landrace population by a haplotype analysis [37]. As low androstenone level was associated with low level of estrone sulphate, they concluded that this haplotype is less desirable for selection purposes because of potentially reduced reproduction.

On SSC 6 the QTL identified to affect androstenone in Duroc overlap the one identified in a Norwegian Duroc population [7]. In order to keep the level of fertility in the population they also investigated how the QTL would affect the level of testosterone and estrogens. They found that most of the QTL shown to affect androsterone also affected one or more of the other hormones except the one identified on SSC 6. In the current study, the fixed effect was found to be 0.775 μg/g and highly significant, and the frequency of the haplotype was rather abundant (56%). An analysis to find the proportion of explained phenotypic variance for the haplotype was conducted. Here it was found that the haplotype explained around 11.7% of the total phenotypic variance. High levels of variance explained for SNPs in a number of different genes have previously been described in a Norwegian Duroc population [37]. As 99.4% of the animals were included in the test and only four haplotypes were identified within this QTL the haplotype appears as a possible candidate for use in breeding efforts.

The QTL identified on SSC 14 between D and androstenone was also confirmed by the haplotype analysis. The haplotype showed a relatively large fixed effect (1.183 μg/g) that explained around 8.1% of the total variance. A QTL analysis conducted in a Meishan × Large White population revealed a genome-wide significant peak within the same area [32]. In the current study the peak SNP was found to be located within an intron of the gene *LOC100518755 *(polypeptide N-acetylgalactosaminyltransferase-like 6-like). Previously, NAT12 (N-acetyltransferase 12) involved in phase II metabolism in the liver has been shown to be differentially expressed when analysing extreme high vs. extreme low androstenone Duroc and Norwegian Landrace animals [35].

The QTL identified in D on SSC 12 that regulates the level of androstenone was also identified in Y and by a single SNP in L. All suggestive QTL were confirmed by the haplotype analysis. In D the most abundant haplotype (51.5%) showed a fixed effect (1.29 μg/g), which was the highest effect on androstenone in the entire study. Further investigation revealed that this particular haplotype explained about 13.2% of the total variance. The fixed effect in Y and L was not as high, 0.827 and 0.945, respectively, and no significant result on the variance could be achieved for these haplotypes.

In L, four additional QTL regions affecting the level of androstenone were confirmed by the haplotype analysis. On SSC 7 a very broad region spanning 38,292 kb was identified. In the study by Grindflek et al. [33], three QTL regions were identified on this chromosome. One of these QTL, referred to as 7a in their paper, overlaps the region found here. However, the region identified in this study also overlaps another QTL previously detected. The *CYP21 *gene was selected as a candidate gene and further analysed, however, they found that no SNP in the coding part of the *CYP21 *gene could explain the association [32]. The *CYP21 *gene is involved in formation of 4,16-androstadien-3-ene from progesterone [2]. In this study we found that the SNP UMB10000108, which was located within an intron of the *CYP21A2 *(cytochrome P450, family 21, subfamily A, polypeptide 2) gene, was associated with the QTL (p = 0.0017). The gene *CYP21 *has previously been shown to be down regulated in high androstenone Norwegian Landrace boars [38].

On SSC 1 in L we identified another suggestive QTL. In this case the analysis of the most common haplotype within the QTL did not show a significant association between the haplotype and trait. However, the second most frequent haplotype seemed to deviate more from the total mean and hence was analysed instead. In this case the haplotype showed a large effect (1.279 μg/g) and it was found that this haplotype explained about 16.8% of the phenotypic variance. A candidate gene located within the region is the gene *CYB5A *(cytochrome b5 type A). Pregnenolone is converted to 5,16-androstadien-3β-ol by the andien-β synthase. The andien-β synthase activity is catalyzed by cytochrome P450c17 and depends on adequate levels of CYB5A [16]. The level of CYB5A in testis hence has an indirect impact on the amount of produced androstenone. A SNP (G > T) at base -8 upstream of ATG in the 5'untranslated region of *CYB5A *was studied in relation to plasma and back fat traits related to boar taint in Swedish Landrace × Yorkshire crossbred pigs [42]. The analysis showed weight dependent positive effect of having the T allele in both plasma androstenone and back fat skatole. However, they concluded that it should not be implemented due to the lack of change in back fat androstenone that might be due to the low allele frequency of the T allele in the population.

One of the QTL identified on SSC 2 to affect androstenone was found to overlap a region affecting skatole reported by Grindflek et al. (2011) [7]. Further investigations of the area will be needed before including this QTL in a breeding scheme.

### Haplotype analysis in relation to the indolic compounds

A total of five QTL verified by the haplotype analysis in relation to indole and the S/I index seemed to be influenced by the slaughter weight as well as the trait. Hence, the haplotype were re-analysed to account for the effects of the slaughter weight. For the three S/I index QTL on SSC 6, 8 and 14 identified in Y, Y and L the fixed effect dropped by 0.011, 0.010 and 0.027 μg/g, respectively, when including the slaughter weight as a covariate. The amount of variance explained by the haplotype was analysed to see how it was affected by including the slaughter weight in the model. Surprisingly, the values were still high; 8.9% in Y on SSC 6a, 7.63% in Y on SSC 8 and 11.21% in L on SSC14, were only the one on SSC 6 had changed markedly (-2.7%). Even though the fixed effect decreased in these cases, the explained variance only changed marginally. The fixed effect of the haplotype identified from the chromosome 10b QTL in D also showed a decrease (0.005 μg/g). This QTL overlap a QTL for both skatole and indole previously identified in the Landrace population [33].

Only two QTL identified to affect skatole were confirmed by the haplotype analysis, namely, the one on SSC 9 in Y and the one in D on SSC 3. The QTL identified in D on SSC 3 overlap a QTL identified to affect both testosterone and esterone sulphate in a Norwegian Landrace population [7]. Besides, they identified a QTL affecting both skatole and androstenone in a Norwegian Duroc population to be located closer to the centromere region than the one identified in this study. In the current study, a suggestive QTL for androstenone was also detected (P = 0.0004). A candidate gene for the QTL on SSC 3 is the *SULT1A1 *(sulfotransferase family, cytosolic, 1A, phenol-preferring, member 1) gene. This gene has not yet been aligned to the porcine assembly, but comparative mapping of human genes within the area of *SULT1A1 *suggests that the gene is likely to be located around 16.6-17.0 Mb on SSC 3 Sscrofa9. In addition, the peak SNP (DIAS0001357) was found to be located within the *XPO6 *gene situated only 0.4 Mb from *SULT1A1 *in humans. Furthermore, the gene has been mapped by linkage analysis to SSC 3 [43]. This gene is known to be involved in phase II metabolism of skatole [23]. In addition, a suggestive QTL on SSC 7 identified to affect skatole in the Y breed that could almost be distinguished by the haplotype analysis (p = 0.088) overlap the region reported in a Norwegian landrace population [7]. This region was also reported by Quintanilla et al. (2003) to affect back fat androstenone [32].

The QTL identified and confirmed by the haplotype analysis on SSC 14 in both L and Y affecting indole, and in L also the S/I index, could be regulated by the candidate gene *CYP2E1 *(cytochrome P450, family 2, subfamily E, polypeptide 1). This gene is a phase I liver metaboliser of skatole (reviewed by Zamaratskaia et al. 2008 [15]). In addition, the *CYP2E1 *gene has previously been shown to be a major metaboliser of indole in rats [44]. The UMB10000045 SNP situated within an intron of the gene was found to be significantly associated with the QTL (p < 1e-5) in L for both traits. However, this SNP was not segregating in the Y population; instead, another SNP was identified in relation to the gene. The SIRI0000194 flanked the *CYP2E1 *at the 5' side of the gene (587 bp) and was found to be significantly association to the QTL (p = 8e-6). For both of the haplotypes identified in relation to indole the fixed effects seemed small (-0.004 and -0.007). The negative values indicate that for the most frequent haplotype in these QTL the average level of indole was higher. By analysing the explained variance for the haplotypes it was found that the level was similar to previously identified values [37]. The variance explained by the haplotype regulating indole in L was 4.2%, but the level identified in Y was much higher: 13.9%. The variance explained by the S/I index in L was 11.5%.

None of the breeds showed any association to the pure skatole measure within the chromosome region on SSC 14. One of the issues that have been discussed during the past decade is the inability to detect a skatole QTL in relation to *CYP2E1 *[2]. The difference in bacterial load in the animals and hence the synthesis of the compounds might explain the lack of QTL. Indole is produced in a single step by a large variety of bacteria whereas the two-step formation of skatole is specific and dependent on certain bacteria strains. The first step from tryptophan to indole-3-acetic acid is done by *Escherichia Coli *and *Clostridium *[45,46]. The second step converts the indole-3-acetic acid into skatole. This is done by the genera *Clostridium *and a *Lactobacillus *strain [47-50]. The genetic composition of an animal does not depend on whether the skatole is produced or not. If a proportion of the animals have less skatole due to a low bacterial load their genetic ability to metabolise the compound can still be rather poor but give a "false" low trait value in relation to their genetics. In addition, indole and skatole are both broken down by CYP2E1 in the liver as described above but as the level of indole is not affected by the composition of bacteria we see a perfect association to the genetics in the area around the gene. The same applies for the skatole equivalent trait. As indole is part of the trait we get a more balanced trait in relation to the genetics. In a study by Lanthier et al. (2007) [51] intestinal skatole were measured to distinguish high and low producers. They found that only the high producers showed a strong correlation between fat skatole and the level of *SULT1A1*. This might be due to the same principle as described above. However, the more recent studies on Norwegian pure bred animals showed association of skatole and indole to affect the genomic region in both Landrace and Duroc [7,33].

## Conclusion

In this study we conducted QTL analysis of four traits related to boar taint in three different Danish breeds. More than half of the suggestive QTL that were identified were confirmed by the haplotype analysis and many of them also by previous findings. A number of candidate genes were identified. In relation to androstenone the genes *SRD5A2*, *LOC100518755*, *CYB5A *and *CYP21A2 *were located within QTL in either Duroc or Landrace. In addition, the genes that might influence the level of skatole and indole within the identified QTL were *SULT1A1*, and *CYP2E1*. Identification of causal variation within the candidate genes will further advance attempts to apply genetic markers to select for reduced boar taint.

## Methods

The analysis conducted in this article deviates from most other approaches where either a SNP or haplotype analysis is conducted. In this case we did both. A classical genome scan, however somewhat sparse, was done to identify QTL regions and in addition, a haplotype analysis to confirm the QTL association as well as identify haplotypes to reduce the level of boar taint. As this analysis was not performed on traditionally haplotype blocks but on highly associated markers, SNPs within the QTL were selected and used in the analysis. The ultimate target was to identify haplotypes that can be applied to selective breeding.

### Animals

The 923 pigs used in the study comprise boars of three breeds. A total of 265 Danish Duroc, 265 Yorkshire (Danish Large white) and 393 Danish Landrace boars were used. All population pedigrees included nuclear families with 1-2 offspring in a half-sib family structure. The Duroc population comprised offspring of 71 boars crossed with 229 sows and includes half sib families of up to 10 animals. The Landrace population comprised offspring of 56 boars crossed with 264 sows where the largest half sib family included 24 offspring. Finally, the Yorkshire population comprised offspring of 51 boars crossed with 179 sows including half sib families of 2-25 animals. The boars were selected based on their potential to be genetically superior for the production traits by virtue of the ancestry and were all performance tested at the test station Bøgildgård. Each boar was bred on one of the 49 DanAvl nucleus breeding farms. The newly arrived boars (approx. 7 kg) were kept in climate stables for the first weeks. When they reached 30 kg they were moved to the testing stables and sorted according to breeds. They were put into pens with ACEMO automatic dry feeding stations, and fed ad libitum from 30 kg to 100 kg live weight with the same feed composition. After end of performance testing they were kept at the station until slaughter. Additional information about the test station can be found in [52]. The boars were slaughtered from week 41 in 2006 to week 20 in 2007 when the largest pig in the pen reached a weight of 110 kg. At slaughter, tissue samples of muscle, liver, testis, and fat were collected and stored at -20°C.

### Trait analysis

Slaughter weight and meat content was determined no later than 45 minutes after slaughter using the standard classification system in Danish slaughterhouses. The automated equipment worked by keeping the carcass fixed while seven injection probes with light reflection measured the fat and meat thickness from the ham, loin and front. By combining these measures, a value for meat content was created. The animal age was registered in weeks. A skatole equivalent containing a combined value of skatole and indole (S/I) was measured by a calorimetric method in adipose tissue in 85 percent of the animals [53]. In addition, skatole, indole and androstenone were measured by the Norwegian School of Veterinary Sciences (NVH) on a subset of the animals (180 D, 259 L and 132 Y). Adipose tissue was examined by high performance liquid chromatography to measure levels of skatole and indole [54]. Levels of androstenone were analysed by modified time-resolved fluoroimmunoassay [55], using an antibody produced at NVH [56]. The trait values were log transformed using log base 10 in order to meet the assumption of Gaussian distributed data.

### Genotyping and data validation

Genomic DNA was isolated from all specimens by treatment with proteinase K followed by sodium chloride precipitation [57]. Single Nucleotide Polymorphisms of all boars were genotyped on the PorcineSNP60 [58] Illumina iSelect BeadChip according to the protocol [59]. Thereafter, data were analysed by the Haploview 4.2 software [60] to account for low minor allele frequency (MAF > 0.05) and deviation from Hardy-Weinberg proportions (p-value > 0.001) [61]. The analysis was performed on one breed at a time as the association analysis did not account for population stratification that would be introduced by analysing the breeds together. Tag SNP sets were selected by Haploview 4.2's implementation of Tagger [39] to generate a less conservative Bonferroni significance level for the association analysis. The SNP containing sequences were then mapped to both the Pig Sscrofa9 release 56 from the Ensembl database [62] and the Pig Sscrofa10.2 recently released [63] (International Swine Genome Sequencing Consortium). The mapping to Pig Sscrofa9 was used for gene annotation.

### Association analysis with SNP effects

The four boar taint traits were analysed in relation to all segregating markers for one breed at a time. Two different tests for evidence of association were performed. The first test was run in a naive way to find evidence of association without taking into account the family structure, seasonal fluctuations, pen, herd, slaughter weight or meat content. This test was performed as a total evidence of association conducted by use of simple F-statistics as implemented in QTDT version 2.5.1 [64]. It was used to test whether the two alleles in a SNP showed any significant difference in mean value. The second test was performed by the PLINK software using the qfam-total option [65]. This test accounted for the nuclear structure, but not the half-sib structure represented by the data. Besides, the trait values in this case were adjusted for season (month) and pen at the test station Bøgildgård, if the trait was found to be influenced by these. The QFAM model was run using the T(max) permutation of 100,000 for all SNPs. All suggestive QTL identified by the two methods significant at the 5% Bonferroni level were selected for further analysis using a mixed model as later described. The body traits (slaughter weight and meat content) were not used as covariates as there might be an overlap in genes affecting both boar taint and the body characters. However, an analysis to identify which of the QTL that affected the body traits was conducted in addition to the haplotype analysis as described below.

### The haplotype algorithm

Considering haplotypes defined from highly associated SNPs, it is possible to account for more of the genetic variance present within the QTL regions, than by single SNPs. Hence, SNP-sets from all QTL regions were manually selected to represent haplotypes based on two criteria. The first criterion was that the SNP at the peak position had to be significant at the 5% Bonferroni chromosome-wide level in at least one of the association analysis performed. The second criterion was based on the degree of association, where the p-values of all the SNPs in the SNP-set should be smaller than the p-value of the background association level. The background level was evaluated by eye within each chromosome for the given trait. If only one SNP had a low p-value this was assigned as a single SNP QTL. The haplotype phases of these SNP-sets were derived probabilistically. As binary trees have previously shown to be excellent for phasing data we used this structure in the algorithm [66]. A recursive algorithm was designed to create a binary tree that contained the frequency of the different possible phases. By ordering the SNPs in the physical order the tree was constructed by starting with the first SNP. At the end-nodes the phase was registered together with a counter representing the probability of having that phase. The counter was updated every time an animal reached the end-node. In the first step all animals within the breed was added creating the population tree. Then, by running through the tree again, it was possible to find the most probable haplotype for each animal given information from the whole population. By subtracting this phase from the genotype in the SNP-set, the second phase was found. To reduce computing time, animals that were heterozygous for all SNPs were not included in the tree construction. Haplotypes having a frequency above 1.5% were considered true haplotype.

### Analysis with fixed haplotype effect

A one-way ANOVA-test implemented in the lme4 package of R was conducted to test if the most common haplotype in the population could account for the QTL. A mixed model was introduced to test the additive effect of the haplotype.

$${\text{Y}}_{\text{i}}=\mu_{\text{f}\left(\text{i}\right)}+{\text{b}}_{\text{f}\left(\text{i}\right)}+{\text{d}}_{\text{nf}\left(\text{i}\right)}+\beta {\text{h}}_{\text{i}}+{\text{s}}_{\text{season}\left(\text{i}\right)}+{\text{p}}_{\text{pen}\left(\text{i}\right)}+{\text{k}}_{\text{herd}\left(\text{i}\right)}+{\text{e}}_{\text{i}}$$

Here Y_i _is the trait value for the individual i, μ_f _is the mean of the half-sib family of individual i, b_f(i) _is the random effect of the boar in the half-sib family of individual i, d_nf(i) _the random effect of the sow in the nuclear family of individual i, and s_season(i)_, p_pen(i) _and K_herd(i) _are the random effect of season, pen and herd for individual i. The term h_i _is h_i _= 1 if two copies of the most common haplotype, 0 if one copy and -1 if zero copies, and the parameter β is the additive genetic effect of the most common haplotype. A significance test for β = 0, i.e. no haplotype effect, was performed.

As described previously, the model did not account for slaughter weight and meat content. Hence these were analysed with the model above for the slaughter weight or meat content. If one or both of these components could also be differentiated by the haplotype the additive mixed model was run again, this time including the body traits as cofactors in the model.

## List of abbreviations

D: Duroc; L: Landrace; Y: Yorkshire; SNP: single nucleotide polymorphism; GWAS: genome wide association study; QTL: quantitative trait locus or loci; S/I index: skatole equivalent containing both skatole and indole; SSC: *Sus scrofa *chromosome; MAS: marker assisted selection; MAF: minor allele frequency; HWP: Hardy-Weinberg proportions.

Genes: *CYB5:CYB5A*: cytochrome b5 type A; *CYP17A1*: cytochrome P450 17A1; *CYP21*:*CYP21A2*: cytochrome P450, family 21, subfamily A, polypeptide 2; *CYP2A*: cytochrome P450, family 2, subfamily A; *CYP2E1*: cytochrome P450, family 2, subfamily E, polypeptide 1; *LOC100518755*: polypeptide N-acetylgalactosaminyltransferase-like 6-like; *SRD5A2: ST5AR2*: steroid 5-alpha-reductase 2 and *SULT1A1*: sulfotransferase family, cytosolic, 1A, phenol-preferring, member 1.

## Authors' contributions

CBE conceived the research and coordinated the project. IHV was in charge of the animal experiment and involved in planning the project. KKS did the SNP genotyping. LNC collected the data and animal tissue samples and critically revised the paper. BG designed the statistical models. VRG validated the data, conducted the GWAS and haplotype analysis as well as drafted the paper. All authors have read, edited and approved the final manuscript.

## Supplementary Material

Additional file 1 **All quantitative trait loci, including SNPs selected for the haplotype analysis, identified using QTDT and PLINK**. The file contains information about breed, trait, SNPs, *Sus scrofa *chromosome and position. Click here for file
